# Comparative effectiveness and safety of oral anticoagulants in patients with atrial fibrillation using antiarrhythmic drugs: An international cohort study

**DOI:** 10.1002/bcp.70391

**Published:** 2025-11-29

**Authors:** Fabian Maximilian Meinert, Jenny Dimakos, Ying Cui, Kristian B. Filion, Christel Renoux, Antonios Douros

**Affiliations:** ^1^ Institute of Clinical Pharmacology and Toxicology Charité‐Universitätsmedizin Berlin Berlin Germany; ^2^ Department of Medicine McGill University Montreal Quebec Canada; ^3^ Centre for Clinical Epidemiology Lady Davis Institute Montreal Quebec Canada; ^4^ Department of Epidemiology, Biostatistics and Occupational Health McGill University Montreal Quebec Canada; ^5^ Department of Neurology and Neurosurgery McGill University Montreal Quebec Canada

**Keywords:** bleeding, drug–drug interaction, ischemic stroke, pharmacoepidemiology

## Abstract

**Aim:**

Our international cohort study assessed the comparative effectiveness and safety of direct oral anticoagulants (DOACs) and vitamin K antagonists (VKAs) among patients with non‐valvular atrial fibrillation (NVAF) using antiarrhythmic drugs.

**Methods:**

Using the United Kingdom's (UK's) Clinical Practice Research Datalink Aurum and Québec claims data, we assembled two study cohorts of patients with NVAF who initiated DOACs or VKAs while on antiarrhythmic drugs (2011–2020). Using an as‐treated exposure definition, we assessed the risks of ischemic stroke and major bleeding associated with DOACs *vs*. VKAs. Cox proportional hazards models estimated hazard ratios (HRs) and 95% confidence intervals (CIs) after propensity score‐based inverse‐probability‐of‐treatment‐weighting. Site‐specific estimates were pooled together using random‐effects models. Secondary analyses assessed effect measure modification by individual DOAC and type of antiarrhythmic drugs (interacting *vs*. non‐interacting).

**Results:**

The study cohort included 44 435 patients with NVAF initiating DOACs (n = 29 071) or VKAs (n = 15 364) while using antiarrhythmics. Compared to VKAs, DOACs were not associated with the risk of ischemic stroke (UK: HR, 0.90; 95% CI, 0.61–1.32/ Quebec: HR, 0.85; 95% CI, 0.70–1.03/ pooled: HR, 0.86; 95% CI 0.72–1.02; I^2^ = 0%) but with a decreased risk of major bleeding (UK: HR, 0.90; 95% CI, 0.75–1.08/Quebec: HR, 0.83; 95% CI, 0.76–0.91/pooled: HR 0.84; 95% CI 0.78–0.92; I^2^ = 0%); the latter was more pronounced with apixaban (pooled HR, 0.71; 95% CI, 0.63–0.81; I^2^ = 74%). There was no effect modification by type of antiarrhythmic drugs.

**Conclusions:**

DOACs were as effective but safer than VKAs among NVAF patients treated with antiarrhythmic drugs.

**What is known about this subject**

- Direct oral anticoagulants (DOACs) are at least effective and safe as vitamin K antagonists (VKAs) in patients with non‐valvular atrial fibrillation.
- Antiarrhythmic drugs as commonly used together with oral anticoagulants in this patient population.

**What this study adds**

- Our large international cohort study showed that DOACs vs VKAs were not associated with the risk of ischemic stroke but with a 16% decrease in the risk of major bleeding.
- The decrease in the risk of major bleeding was more pronounced with apixaban.

## 1 INTRODUCTION

Direct oral anticoagulants (DOACs) have shown similar efficacy and improved safety when compared to the therapeutic alternatives vitamin K antagonists (VKAs) among patients with non‐valvular atrial fibrillation (NVAF).^1^, ^2^ However, the comparative effects of oral anticoagulants are less clear in certain clinically important subgroups such as patients who are additionally treated with antiarrhythmic drugs, a heterogenous group of medications mostly used in patients with NVAF and other arrhythmias for rhythm and rate control.^3^

In NVAF, antiarrhythmic drugs are often used in patients with more permanent patterns of arrhythmia,^4^, ^5^ a strong independent risk factor for ischemic stroke.^6^ Therefore, patients with NVAF requiring both anticoagulation and treatment with antiarrhythmic drugs could potentially be at a higher risk of adverse clinical outcomes than the overall NVAF population. Pharmacokinetic interactions between oral anticoagulants and several antiarrhythmics mediated by the inhibition of the cytochrome P‐450 3A4 (CYP3A4) enzyme and the permeability glycoprotein (p‐pg) transporter could further complicate the management of this population.^7^, ^8^, ^9^, ^10^

Two post‐hoc analyses of randomized trials have assessed the comparative effectiveness and safety of oral anticoagulants among patients with NVAF treated with antiarrhythmic drugs.^11^, ^12^ However, both studies considered use of antiarrhythmic drugs at baseline only, possibly introducing exposure misclassification and depletion of susceptibles. Moreover, they had limited statistical power. Observational studies in the area were restricted to DOAC users,^13^, ^14^, ^15^ focused on specific antiarrhythmic drugs,^13^, ^14^ were affected by selection bias due to inclusion of prevalent users or informative censoring,^14^, ^15^ or had limited statistical power.^13^ Hence, to address the remaining knowledge gap, we conducted an international cohort study comparing the effectiveness and safety between DOACs and VKAs among patients with NVAF treated with antiarrhythmic drugs.

## 2 METHODS

### 2.1 Data source

Our international, population‐based cohort study was conducted using the Clinical Practice Research Datalink (CPRD) Aurum from the United Kingdom (UK) and provincial administrative healthcare databases (the *Régie de l'assurance maladie du Québec*; RAMQ) from Quebec, Canada. The CPRD is the largest primary care database in the world containing records of 60 million patients (18 million patients currently registered) seen across >2000 general practices, and it is representative of the general UK population.^16^, ^17^ In the CPRD, all prescriptions issued by general practitioners are recorded. Moreover, the CPRD contains clinical measures (e.g., blood pressure [BP]), laboratory test results, anthropometric measures (e.g. body mass index [BMI]), and lifestyle variables (e.g. smoking, alcohol consumption).^16^ Patient diagnoses are also recorded in the CPRD and coded using a combination of SNOMED Clinical terms (a structured clinical vocabulary for use in electronic health records) and local EMIS® Web codes (a coding system including clinical events, online test requests, test results and prescriptions), two systems with a greater granularity than the International Classification of Diseases (ICD).^17^, ^18^ The CPRD was linked to the Hospital Episode Statistics (HES) and Office for National Statistics (ONS) databases. The HES includes information on hospital admissions, procedures and discharge diagnoses (coded using ICD‐10). The ONS includes vital statistics data such as date, place and underlying cause of death (coded using ICD‐10) for all citizens living in the UK. The RAMQ covers all residents of the Canadian province of Québec aged ≥ 65 years, those with no private insurance plans, and recipients of financial assistance. The data include demographic characteristics, outpatient diagnoses (coded using ICD‐9 and ICD‐10), procedures and dispensed drug prescriptions. The RAMQ was linked to the *Maintenance et exploitation des données pour l'étude de la clientèle hospitalière* (MED‐ÉCHO) and the *Institut de la statistique du Québec* (ISQ) databases. The MED‐ÉCHO includes information on hospital admissions, procedures and discharge diagnoses (coded using ICD‐10). The ISQ includes vital statistics data.^19^ The independent Scientific Advisory Committee of the CPRD, the data custodian of the RAMQ, and the Research Ethics Board of the Jewish General Hospital Montreal, Canada, approved the study protocol (23_002915).

### 2.2 Study cohort

The source population comprised all patients with a new diagnosis of atrial fibrillation between January 1, 2011 (when dabigatran was the first DOAC approved for the prevention of ischemic stroke among patients with NVAF in the UK and in Canada) and June 22, 2020 (latest date of data availability in the CPRD) or December 12, 2020 (latest date of data availability in the RAMQ). We excluded patients 1) who were younger than 18 years of age; 2) who had a medical history in the database of less than 365 days; 3) who had valvular heart disease diagnosed at any time before the diagnosis of atrial fibrillation (to restrict to NVAF); or 4) who had hyperthyroidism diagnosed in the year before the diagnosis of atrial fibrillation.

From this source population, we included all patients who initiated a DOAC (apixaban, dabigatran, edoxaban, rivaroxaban) or a VKA after the diagnosis of NVAF. We then excluded patients with a prescription for an oral anticoagulant in the year prior (to restrict to new users). We did not exclude patents with prior strokes or prior bleedings to maximize external validity. Finally, we assembled a study cohort that comprised patients with NVAF who initiated DOACs or VKAs and had an overlapping prescription for antiarrhythmic drugs. Antiarrhythmic drugs included sodium channel blockers (flecainide, propafenone), potassium channel blockers (amiodarone, dronedarone, sotalol) and calcium channel blockers (verapamil, diltiazem). We did not consider beta blockers or digoxin because of the heterogeneity of indications of these drugs.^20^, ^21^ Patients were allowed to start antiarrhythmic drugs either prior to or on the same day of the initiation of their oral anticoagulants. Study cohort entry was the date of the initiation of oral anticoagulants. Patients were followed until an event (defined below), treatment discontinuation or switch (defined below), administrative censoring, death or the end of the study period, whichever occurred first. Each patient was allowed to contribute only one episode of concomitant use to the study. An illustration of the study design is provided in Figure S1.

### 2.3 Exposure definition

We used an as‐treated exposure definition, where patients were considered continuously exposed to both drugs if the prescription durations of the drugs of interest (DOACs and antiarrhythmic drugs *vs*. VKAs and antiarrhythmic drugs) were overlapping. We allowed for a 30‐day grace period in the event of non‐overlapping successive prescriptions.

### 2.4 Outcome definition

The primary effectiveness outcome was ischemic stroke, defined as a composite endpoint including hospitalization with ischemic stroke, transient ischemic attack (TIA) or systemic embolism (SE). The primary safety outcome was major bleeding, defined as hospitalization with bleeding. All relevant ICD codes can be found in Table S1.

### 2.5 Covariates

For the analyses on both study outcomes, we adjusted for the following potential confounders measured at cohort entry: age (modelled flexibly using restricted cubic splines), sex assigned at birth, alcohol‐related disorders, arterial hypertension, prior ischemic stroke or TIA, congestive heart failure, coronary artery disease, peripheral vascular disease, major bleeding, type 2 diabetes, liver disease and renal disease, all diagnosed at any time before cohort entry. We also considered cancer (other than non‐melanoma skin cancer) diagnosed in the year before cohort entry. Moreover, we adjusted for the use of antiplatelet agents and selective serotonin reuptake inhibitors in the year before cohort entry. Finally, we included the number of hospitalizations in the year before cohort entry as a proxy for overall health status.

In the CPRD, we additionally included smoking status (current, former, never, unknown), BMI category (<25 kg/m^2^, 25–29 kg/m^2^, ≥30 kg/m^2^, unknown), BP levels (systolic BP ≥ 130 mmHg or diastolic BP ≥ 80 mmHg, systolic BP < 130 mmHg and diastolic BP < 80 mmHg, unknown) and the Index of Multiple Deprivation (quintiles), which is an indicator of socioeconomic status. For all these covariates, we used the last measurement before cohort entry. Moreover, we included estimated glomerular filtration rate (<90 mL/min per 1,73 m^2^) to define renal disease in addition to diagnostic codes. In the RAMQ, we additionally included the number of non‐anticoagulant drugs in the year before cohort entry. Finally, for the analyses on major bleeding, we additionally adjusted for use of non‐steroidal anti‐inflammatory drugs (NSAIDs), proton pump inhibitors and H_2_ blockers, all measured in the year before cohort entry.

### 2.6 Statistical analyses

We calculated crude incidence rates with 95% confidence intervals (CIs) for the study outcomes for each exposure group assuming a Poisson distribution. To minimize potential confounding, we used inverse‐probability‐of‐treatment‐weighting (IPTW) based on propensity scores (PS). PS was calculated via multivariable logistic regression that estimated the probability of receiving the exposure of interest (DOACs) *vs*. the reference category (VKA), conditional on all previously listed covariates. Imbalances in covariates post IPTW were assessed using standardized differences; covariates with standardized differences ≥ 0.1 after weighting were included in the outcome models. We calculated PS separately for different strata defined based on the type of antiarrhythmic drug use at cohort entry (new *vs*. prevalent use) and then pooled the strata‐specific PS. We used Cox proportional hazards models to estimate hazard ratios (HRs) and 95% CIs of the study outcomes and pooled site‐specific estimates together using DerSimonian and Laird random‐effects models.^22^ We used I^2^ to estimate the amount of between‐site heterogeneity that was present.

### 2.7 Secondary analyses

We conducted eight pre‐specified secondary analyses. First, we stratified by subtype of major bleeding (intracranial haemorrhage, gastrointestinal bleeding, other major bleeding). Second, we stratified exposure by individual DOACs (feasible for rivaroxaban and apixaban). Third, we stratified by age (≥70 years, <70 years) and sex assigned at birth (male, female). Fourth, we stratified by baseline risk of the outcome using the CHA_2_DS_2_‐VASc (congestive heart failure, hypertension, age ≥75 years, diabetes mellitus, stroke, vascular disease, age 65–74 years, sex) score (ischemic stroke only) and a modified version of the HAS‐BLED (hypertension, abnormal renal or liver function, stroke, bleeding, elderly, drugs or excess alcohol use) score (major bleeding only). Finally, we stratified by type of antiarrhythmic drug use at cohort entry (new *vs*. prevalent use), the indication for antiarrhythmic drug use (rate control [diltiazem, verapamil] *vs*. rhythm control [amiodarone, dronedarone, flecainide, propafenone, sotalol]) and the CYP3A4‐inhibiting potential of antiarrhythmic drugs (no or weak inhibition [propafenone, flecainide, sotalol] *vs*. moderate inhibition [verapamil, diltiazem, dronedarone, amiodarone]).

### 2.8 Sensitivity analyses

We also conducted seven pre‐specified sensitivity analyses to address different potential sources of bias. First, we used a 15‐day grace period between non‐overlapping successive prescriptions to assess potential exposure misclassification. Second, we used a stricter outcome definition based on hospitalization codes in the primary position only to assess potential outcome misclassification. Third, we additionally included fatal events in the definition of the study outcomes. Fourth, we excluded patients with a history of ischemic stroke or major bleeding to eliminate residual confounding due to prior events. Fifth, we used multiple imputation for missing values for BMI and BP in the CPRD. Sixth, we used an intention‐to‐treat exposure definition imposing a maximum follow‐up of 1 year to address potential selection bias due to informative censoring. Finally, we used inverse‐probability‐of‐censoring‐weighting (IPCW) as an alternate approach to address potential informative censoring, including alcohol‐related disorders, use of antiplatelet agents and NSAIDs (major bleeding only) as time‐varying covariates. Extreme weights were truncated using the 99th percentile as cut‐off. All analyses were conducted with SAS 9.4 software (SAS Institute, Cary, NC).

### 2.9 Nomenclature of targets and ligands

Key protein targets and ligands in this article are hyperlinked to corresponding entries in http://www.guidetopharmacology.org, the common portal for data from the IUPHAR/BPS Guide to PHARMACOLOGY and permanently archived in the Concise Guide to PHARMACOLOGY 2021/22.^23^

## 3 RESULTS

**FIGURE 1:**
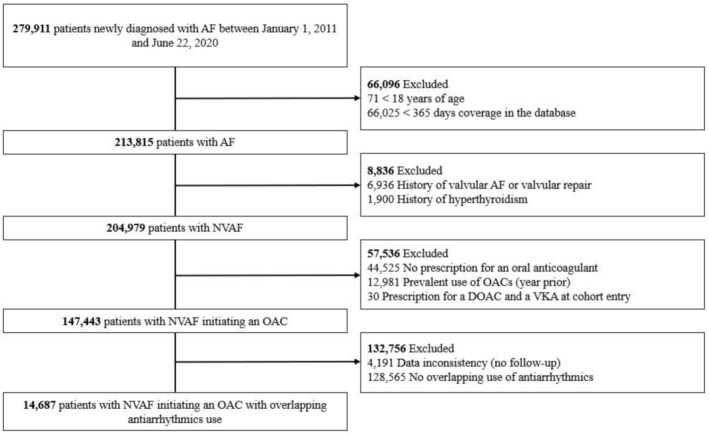
*Flowchart illustrating the construction of the study cohort in the CPRD.* Abbreviations: AF, atrial fibrillation; CPRD, Clinical Practice Research Datalink; NVAF, non‐valvular atrial fibrillation; OAC, oral anticoagulant; DOAC, direct oral anticoagulant; VKA, vitamin K antagonist.

**FIGURE 2:**
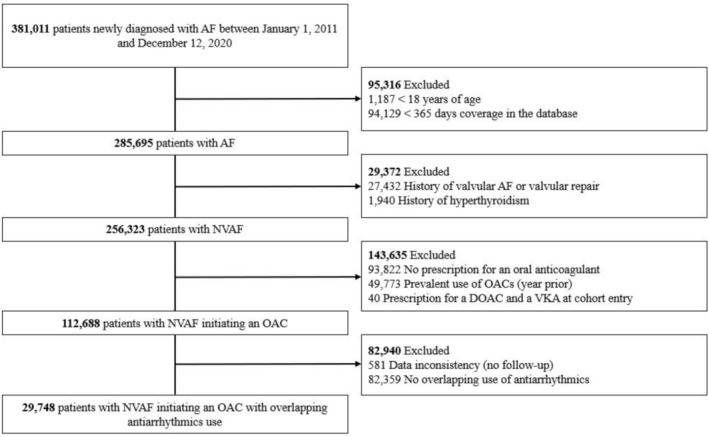
*Flowchart illustrating the construction of the study cohort in the RAMQ.* Abbreviations: AF, atrial fibrillation; RAMQ, Régie de l'assurance maladie du Québec; NVAF, non‐valvular atrial fibrillation; OAC, oral anticoagulant; DOAC, direct oral anticoagulant; VKA, vitamin K antagonist.

The study cohort included44 435 patients with NVAF who initiated DOACs (n = 29 071) or VKAs (n = 15 364) while on antiarrhythmics (Figures 1, 2). Of those, 14 687 patients were in the CPRD and 29 748 patients were in the RAMQ. Patient baseline characteristics are shown in Tables S2‐S3. Overall, patients in the RAMQ were older and had more comorbidities than in the CPRD. Patient characteristics were similar between DOAC users and VKA users in both databases; however, use of antiplatelets agents was more common among VKA users than DOAC users. After IPTW, all baseline characteristics were well balanced.

The results for the primary analyses are shown in Table 1. Compared to VKAs, DOACs were not associated with the risk of ischemic stroke (CPRD: HR, 0.90; 95% CI, 0.61 to 1.32/RAMQ: HR, 0.85; 95% CI, 0.70 to 1.03/ pooled: HR, 0.86; 95% CI, 0.72 to 1.02; I^2^ = 0%). However, there was an association with a decreased risk of major bleeding (CPRD: HR, 0.90; 95% CI, 0.75 to 1.08/Quebec: HR, 0.83; 95% CI, 0.76 to 0.91/pooled: HR, 0.84; 95% CI, 0.78 to 0.92; I^2^ = 0%).

**TABLE 1:** Risk of ischemic stroke and major bleeding associated with use of DOACs *vs.* use of VKAs among patients with NVAF on antiarrhythmic drugs.

	N Patients	N Events	N PY	IR*	Crude HR (95%CI)	IPTW HR (95%CI)	Pooled HR (95% CI)	I^2^
**Ischemic stroke/TIA/SE**							0.86 (0.72–1.02)	0%
**CPRD**								
DOACs	7896	49	6369	7.69	0.94 (0.64–1.39)	0.90 (0.61–1.32)		
VKAs	6791	54	7243	7.46	1.00 (reference)	1.00 (reference)		
**RAMQ**								
DOACs	21 175	377	32 408	11.63	0.70 (0.58–0.84)	0.85 (0.70–1.03)		
VKAs	8573	155	8203	18.90	1.00 (reference)	1.00 (reference)		
**Major bleeding**							0.84 (0.78–0.92)	0%
**CPRD**								
DOACs	7896	236	6250	37.76	0.97 (0.81–1.16)	0.90 (0.75–1.08)		
VKAs	6791	256	7045	36.34	1.00 (reference)	1.00 (reference)		
**RAMQ**								
DOACs	21 175	1569	31 393	49.98	0.67 (0.61–0.74)	0.83 (0.76–0.91)		
VKAs	8573	664	7881	84.26	1.00 (reference)	1.00 (reference)		

* IR per 1000 PY.

Abbreviations: PY, patient years; IR, incidence rate; HR, hazard ratio; CI, confidence interval; IPTW, inverse probability of treatment weighting; DOACs, direct oral anticoagulants; VKAs, vitamin K antagonists; NVAF, non‐valvular atrial fibrillation; TIA, transient ischemic attack; SE, systemic embolism; CPRD, Clinical Practice Research Datalink; RAMQ, Régie de l'Assurance‐Maladie du Québec.

When stratifying by major bleeding subtype, there was an association with a decreased risk of intracranial haemorrhage (pooled HR, 0.53; 95% CI, 0.38 to 0.74; I^2^ = 0%) and a decreased risk of other major bleeding (pooled HR, 0.85; 95% CI, 0.76 to 0.95; I^2^ = 0%) but not with the risk of gastrointestinal bleeding (pooled HR, 0.90; 95% CI, 0.79 to 1.02; I^2^ = 0%) (Table 2). The results of the other secondary analyses are shown in Tables S4‐S9. There were no major effect measure modifications by sex, or type, indication and CYP3A4‐inhibiting potential of antiarrhythmic drug use. However, the decrease in the risk of major bleeding with DOACs was stronger among patients aged less than 70 years (pooled HR, 0.69; 95% CI, 0.58 to 0.82; I^2^ = 57%) than among patients aged 70 years or older (pooled HR, 0.90; 95% CI, 0.82 to 0.98; I^2^ = 0%). Moreover, apixaban showed overall more favourable findings both for ischemic stroke (apixaban: pooled HR, 0.67; 95% CI, 0.53 to 0.86; I^2^ = 0% / rivaroxaban: pooled HR, 0.94; 95% CI, 0.76 to 1.16; I^2^ = 0%) and major bleeding (apixaban: pooled HR, 0.71; 95% CI, 0.63 to 0.81; I^2^ = 74% /rivaroxaban: pooled HR, 1.02; 95% CI, 0.92 to 1.13; I^2^ = 0%) than rivaroxaban.

**TABLE 2:** Risk of different subtypes of major bleeding associated with the use of DOACs *vs.* use of VKAs among patients with NVAF on antiarrhythmic drugs.

	N Patients	N Events	N PY	IR*	Crude HR (95%CI)	IPTW HR (95%CI)	Pooled HR (95% CI)	I^2^
**Intracranial haemorrhage**							0.53 (0.38–0.74)	0%
**CPRD**								
DOACs	7896	7	6396	1.09	0.62 (0.24–1.58)	0.64 (0.25–1.64)		
VKAs	6791	12	7265	1.65	1.00 (reference)	1.00 (reference)		
**RAMQ**								
DOACs	21 175	84	32 643	2.57	0.45 (0.32–0.64)	0.52 (0.37–0.74)		
VKAs	8573	50	8265	6.05	1.00 (reference)	1.00 (reference)		
**Gastrointestinal bleeding**							0.90 (0.79–1.02)	0%
**CPRD**								
DOACs	7896	90	6341	14.19	1.11 (0.82–1.49)	0.96 (0.71–1.29)		
VKAs	6791	84	7207	11.66	1.00 (reference)	1.00 (reference)		
**RAMQ**								
DOACs	21 175	697	32 117	21.70	0.71 (0.62–0.82)	0.88 (0.77–1.02)		
VKAs	8573	285	8101	35.18	1.00 (reference)	1.00 (reference)		
**Other major bleeding**							0.85 (0.76–0.95)	0%
**CPRD**								
DOACs	7896	147	6302	23.33	0.93 (0.74–1.16)	0.89 (0.71–1.12)		
VKAs	6791	169	7098	23.81	1.00 (reference)	1.00 (reference)		
**RAMQ**								
DOACs	21 175	892	31 865	27.99	0.68 (0.61–0.77)	0.84 (0.74–0.95)		
VKAs	8573	370	8042	46.01	1.00 (reference)	1.00 (reference)		

* IR per 1000 PY.

Abbreviations: PY, patient years; IR, incidence rate; HR, hazard ratio; CI, confidence interval; IPTW, inverse probability of treatment weighting; DOACs, direct oral anticoagulants; VKAs, vitamin K antagonists; NVAF, non‐valvular atrial fibrillation; TIA, transient ischemic attack; SE, systemic embolism; CPRD, Clinical Practice Research Datalink; RAMQ, Régie de l'Assurance‐Maladie du Québec.

**FIGURE 3:**
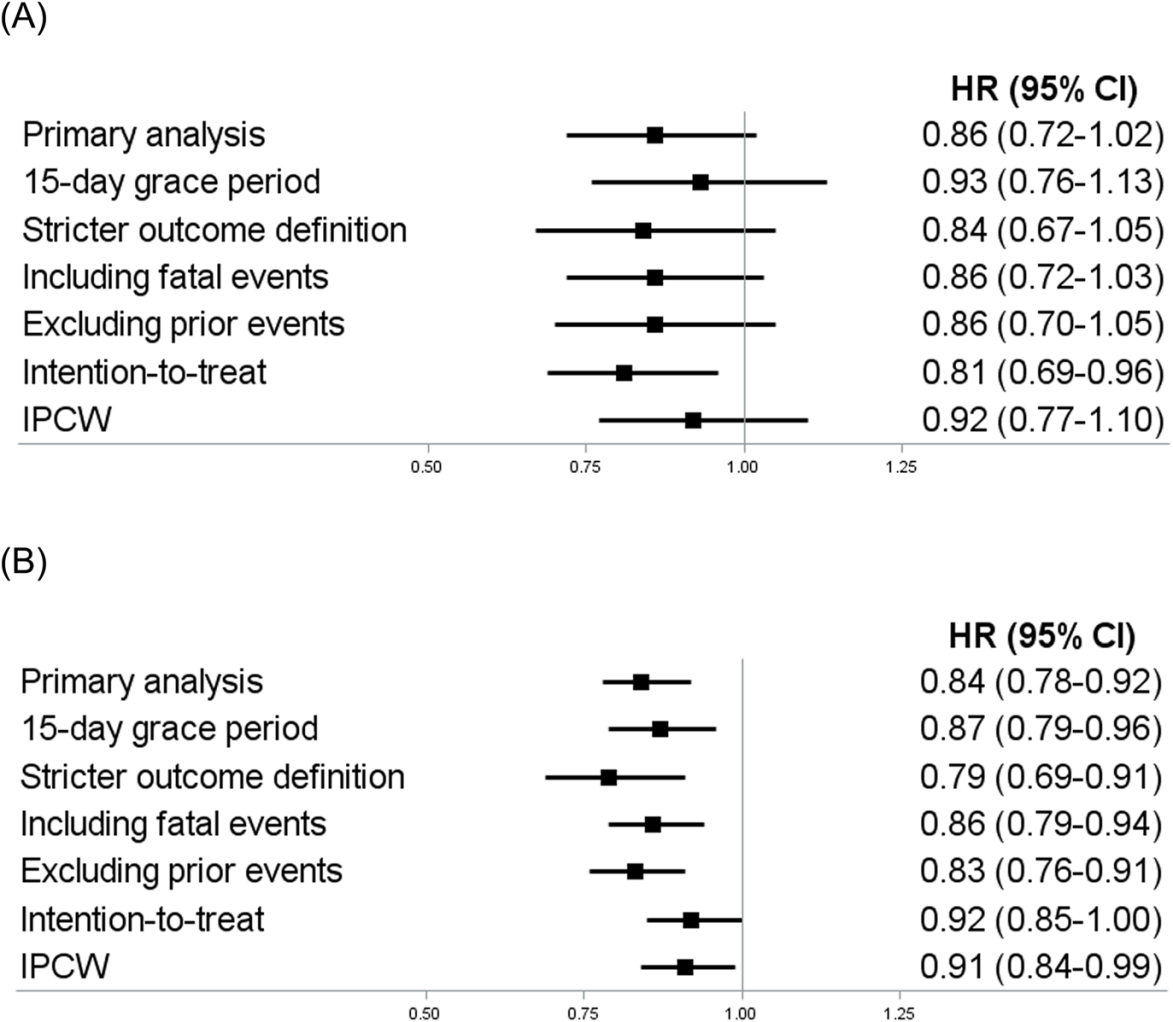
*Forest plots summarizing sensitivity analyses for both study outcomes. Panel a: ischemic stroke. Panel B: major bleeding.* Abbreviations: HR, hazard ratio; CI, confidence interval; IPCW, inverse probability of censoring weights.

The results of most sensitivity analyses were consistent with those of the primary analyses (findings are presented in detail in Tables S10‐S11 and summarized in Figure 3). However, in analyses addressing potential informative censoring, the decrease in the risk of major bleeding observed with DOACs was attenuated (intention‐to‐treat: pooled HR, 0.92; 05% CI, 0.85 to 1.00; I^2^ = 0% /IPCW: pooled HR, 0.91; 0.84 to 0.99; I^2^ = 0%).

## 4 DISCUSSION

Our large, international cohort study showed that compared with VKAs, DOACs were not associated with the risk of stroke but with a decreased risk of major bleeding among patients with NVAF who were treated with antiarrhythmic drugs. The decreased risk of major bleeding was driven by decreased risks in intracranial and other non‐gastrointestinal major bleedings, it was more pronounced among patients aged less than 70 years, and it was only observed with apixaban but not with rivaroxaban. In sensitivity analyses exploring potential informative censoring, though, the decreased risk was attenuated.

Our results showing overall more favourable effects with apixaban than rivaroxaban among patients with NVAF requiring both oral anticoagulation and antiarrhythmic drug treatment are in line with previous post‐hoc analyses of randomized trials.^11^, ^12^ In a post‐hoc analysis of the ARISTOTLE trial that was restricted to patients exposed to amiodarone at baseline, apixaban was associated with a numerical decrease in the risk of stroke (HR, 0.68; 95% CI, 0.40 to 1.15) and with a decreased risk of major bleeding (HR, 0.61; 95% CI, 0.39 to 0.96), when compared to warfarin.^11^ In contrast, in a similar post‐hoc analysis of ROCKET‐AF, rivaroxaban was associated with numerical increases in the risks of stroke (HR, 1.71; 95% CI, 0.80 to 3.65) and major bleeding (HR, 2.20; 95% CI, 0.98 to 4.91), when compared to warfarin.^12^

Potential explanations for the effect heterogeneity with respect to the risk of major bleeding between apixaban and rivaroxaban among users of antiarrhythmic drugs should include both pharmacodynamic and pharmacokinetic considerations. On one hand, while both apixaban and rivaroxaban are direct factor Xa inhibitors, the former has shown a more favourable safety profile than the latter in the overall NVAF population in RCTs^24^ and in observational studies.^25^ On the other hand, both apixaban and rivaroxaban are CYP3A4 and p‐gp substrates, and concomitant use of each of the two DOACs with strong CYP3A4 or p‐gp inhibitors or inducers has led to similar changes in their systemic levels.^26^ Therefore, pharmacokinetic interactions between apixaban or rivaroxaban and certain CYP3A4 and p‐gp inhibiting antiarrhythmic drugs do not seem to play a major role in the observed effects.

We observed no differences in the risk of ischemic stroke and a moderately decreased risk of major bleeding with DOACs *vs*. VKAs. Overall, these findings are consistent with those from the randomized trials in the overall NVAF population. In a meta‐analysis of the four major trials in the area, DOACs were not associated with the risk of ischemic stroke (risk ratio, 0.92; 95% CI, 0.83–1.02) but with a numerical decrease in the risk of major bleeding (risk ratio, 0.86; 95% CI, 0.73–1.00), when compared to warfarin.^24^ Therefore, it seems that the comparative effectiveness and safety of DOACs *vs*. VKAs that have been extensively described in the overall NVAF population is retained among the high‐risk group of patients with NVAF who are additionally treated with antiarrhythmic drugs.

Our study has several strengths. First, we had a large sample size that allowed the estimation of precise effect estimates for both study outcomes. Moreover, a high level of precision was also possible in several secondary analyses assessing potential effect measure modification with specific compounds or in clinically important subgroups. Second, the utilization of two databases from two different countries and healthcare systems likely maximized the external validity of our findings. Third, the use of a common study protocol in both databases provided a high level of methodological homogeneity, allowing us to pool together the site‐specific findings.

Our study has also some limitations. First, given that treatment duration was defined based on prescriptions (in the CPRD) or dispensations (in the RAMQ) and not on actual medication use, exposure misclassification is possible. However, a sensitivity analysis using a shorter grace period yielded consistent findings. Second, outcome misclassification is also possible. To address the potential impact of this bias, we performed two sensitivity analyses either using a stricter outcome definition, where we considered hospitalization codes in primary position only, or expanding our outcome definition by additionally including fatal events; both analyses were consistent with the primary analysis. Third, despite the use of PS‐based IPTW that included many potential confounders, residual confounding cannot be excluded given the observational design. Finally, our analyses did not consider whether the dose of DOACs was properly adjusted based on the level of renal function.

Overall, our international cohort study suggests that DOACs are at least as effective and safer than VKAs among patients with NVAF using antiarrhythmic drugs. Our study also alludes to more favourable effects with apixaban than with rivaroxaban. Hence, in congruence with current guidelines for the overall NVAF population, DOACs could also be recommended for the high‐risk group of patients who require additional treatment with antiarrhythmic drugs. Moreover, while the beneficial effects of apixaban in this setting should be corroborated in future studies comparing head‐to‐head different DOACs, our results support preferential prescribing of apixaban.

## AUTHOR CONTRIBUTIONS

FMM drafted the manuscript. JD critically revised the manuscript. YC conducted the statistical analyses. KBF and CR critically revised the manuscript. AD designed the study, supervised the study and critically revised the manuscript.

## CONFLICT OF INTEREST STATEMENT

The authors have no conflicts of interest to declare.

## Supporting information


**Figure S1.** Study design.
**Table S1.** ICD‐10 codes for the definition of ischemic stroke and major bleeding.
**Table S2.** Baseline characteristics of patients in the CPRD.
**Table S3.** Baseline characteristics of patients in the RAMQ.
**Table S4.** Risk of ischemic stroke associated with use of DOACs compared with use of VKAs among patients with NVAF treated with antiarrhythmics (stratification by demographics).
**Table S5.** Risk of ischemic stroke associated with use of DOACs compared with use of VKAs among patients with NVAF treated with antiarrhythmics (stratification by baseline risk and individual DOACs).
**Table S6.** Risk of ischemic stroke associated with use of DOACs compared with use of VKAs among patients with NVAF treated with antiarrhythmics (stratification by types of antiarrhythmics).
**Table S7.** Risk of major bleeding associated with use of DOACs compared with use of VKAs among patients with NVAF treated with antiarrhythmics (stratification by demographics).
**Table S8.** Risk of major bleeding associated with use of DOACs compared with use of VKAs among patients with NVAF treated with antiarrhythmics (stratification by baseline risk and individual DOACs).
**Table S9.** Risk of major bleeding associated with use of DOACs compared with use of VKAs among patients with NVAF treated with antiarrhythmics (stratification by types of antiarrhythmics).
**Table S10.** Risk of ischemic stroke associated with use of DOACs compared with use of VKAs among patients with NVAF treated with antiarrhythmics (sensitivity analyses).
**Table S11.** Risk of major bleeding associated with use of DOACs compared with use of VKAs among patients with NVAF treated with antiarrhythmics (sensitivity analyses).

## Data Availability

The data that support the findings of this study are available from the data custodians of the UK CPRD and the RAMQ. Restrictions apply to the availability of these data, which were used under licence for this study.
