# Supplementary material for: Comparative effectiveness and safety of oral anticoagulants in patients with atrial fibrillation using antiarrhythmic drugs: An international cohort study
Source: Br J Clin Pharmacol. 2025 Nov 29;92(4):1177–85. doi: 10.1002/bcp.70391 (PMC13021287; doi:10.1002/bcp.70391)
Supplement: Supplementary file 1 — Figure S1. Study design. Table S1. ICD‐10 codes for the definition of ischemic stroke and major bleeding. Table S2. Baseline characteristics of patients in the CPRD. Table S3. Baseline characteristics of patients in the RAMQ. Table S4. Risk of ischemic stroke associated with use of DOACs compared with use of VKAs among patients with NVAF treated with antiarrhythmics (stratification by demographics). Table S5. Risk of ischemic stroke associated with use of DOACs compared with use of VKAs among patients with NVAF treated with antiarrhythmics (stratification by baseline risk and individual DOACs). Table S6. Risk of ischemic stroke associated with use of DOACs compared with use of VKAs among patients with NVAF treated with antiarrhythmics (stratification by types of antiarrhythmics). Table S7. Risk of major bleeding associated with use of DOACs compared with use of VKAs among patients with NVAF treated with antiarrhythmics (stratification by demographics). Table S8. Risk of major bleeding associated with use of DOACs compared with use of VKAs among patients with NVAF treated with antiarrhythmics (stratification by baseline risk and individual DOACs). Table S9. Risk of major bleeding associated with use of DOACs compared with use of VKAs among patients with NVAF treated with antiarrhythmics (stratification by types of antiarrhythmics). Table S10. Risk of ischemic stroke associated with use of DOACs compared with use of VKAs among patients with NVAF treated with antiarrhythmics (sensitivity analyses). Table S11. Risk of major bleeding associated with use of DOACs compared with use of VKAs among patients with NVAF treated with antiarrhythmics (sensitivity analyses). [file BCP-92-1177-s001.docx]

**Contents**

[eFigure 1. Study design 2](#_Toc211533494)

[eTable 1. ICD-10 codes for the definition of ischemic stroke and major bleeding 3](#_Toc211533495)

[eTable 2. Baseline characteristics of patients in the CPRD 4](#_Toc211533496)

[eTable 3. Baseline characteristics of patients in the RAMQ 7](#_Toc211533497)

[eTable 4. Risk of ischemic stroke associated with use of DOACs compared with use of VKAs among patients with NVAF treated with antiarrhythmics (stratification by demographics) 9](#_Toc211533498)

[eTable 5. Risk of ischemic stroke associated with use of DOACs compared with use of VKAs among patients with NVAF treated with antiarrhythmics (stratification by baseline risk and individual DOACs) 11](#_Toc211533499)

[eTable 6. Risk of ischemic stroke associated with use of DOACs compared with use of VKAs among patients with NVAF treated with antiarrhythmics (stratification by types of antiarrhythmics) 13](#_Toc211533500)

[eTable 7. Risk of major bleeding associated with use of DOACs compared with use of VKAs among patients with NVAF treated with antiarrhythmics (stratification by demographics) 15](#_Toc211533501)

[eTable 8. Risk of major bleeding associated with use of DOACs compared with use of VKAs among patients with NVAF treated with antiarrhythmics (stratification by baseline risk and individual DOACs) 17](#_Toc211533502)

[eTable 9. Risk of major bleeding associated with use of DOACs compared with use of VKAs among patients with NVAF treated with antiarrhythmics (stratification by types of antiarrhythmics) 19](#_Toc211533503)

[eTable 10. Risk of ischemic stroke associated with use of DOACs compared with use of VKAs among patients with NVAF treated with antiarrhythmics (sensitivity analyses) 21](#_Toc211533504)

[eTable 11. Risk of major bleeding associated with use of DOACs compared with use of VKAs among patients with NVAF treated with antiarrhythmics (sensitivity analyses) 23](#_Toc211533505)

# **eFigure 1. Study design**


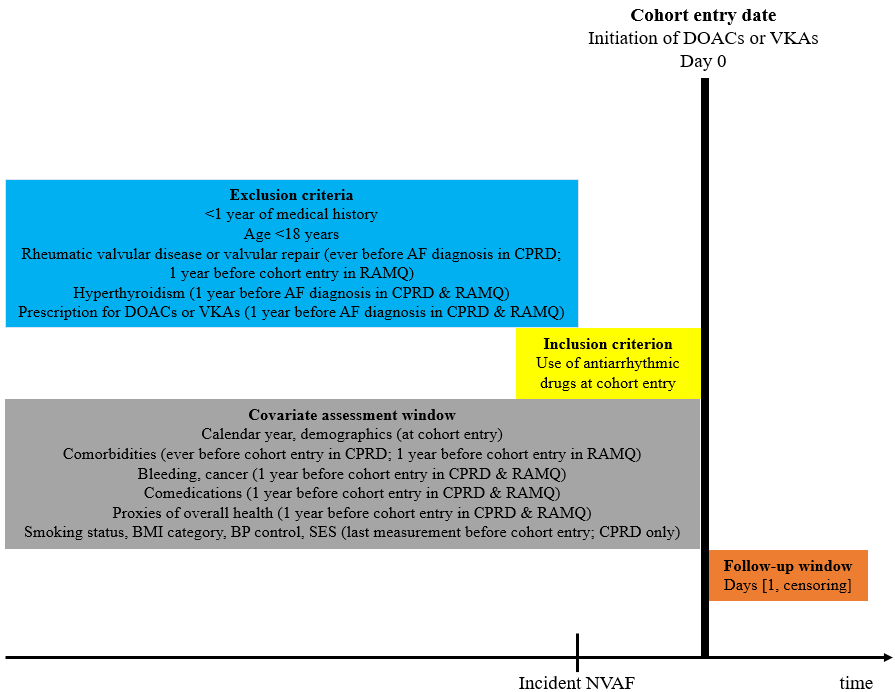


Abbreviations: CPRD, Clinical Practice Research Datalink; RAMQ, Régie de l'Assurance-Maladie du Québec; NVAF, non-valvular atrial fibrillation; BMI, body mass index; BP, blood pressure; SES, socioeconomic status.

# **eTable 1. ICD-10 codes for the definition of ischemic stroke and major bleeding**

| **Outcomes** | **ICD-10 code** |
| --- | --- |
| **Ischemic stroke / TIA / Systemic embolism** | I63, I64, H34.1, G45, H340, I74 |
| **Major bleeding (Intracranial bleeding)** | I60, I61, I621, I620, I629 |
| **Major bleeding (Gastrointestinal bleeding)** | I850, K920, K921, K922, K2211, K226, K228, K250, K252, K254, K256, K260, K262, K264, K266, K270, K272, K274, K276, K280, K282, K284, K286, K290, K294, K2921, K2961, K2971, K2991, K2981, K3181, K5711, K5713, K5731, K5733, K661, K625, K552 |
| **Major bleeding (Other major bleeding)** | D699, H0289, H0523, H113, H313, H356, H431, H44819, I230, I312, J94.2, M250, M7981, N02, N3289, N92, N950, N837, N939, R040, R041, R042, R048, R049, R233, R31, R58, T792, T810 |

Abbreviations: ICD-10, International Classification of diseases, 10^th^ Revision; TIA, transient ischemic attack.

# **eTable 2. Baseline characteristics of patients in the CPRD**

|  | **Before IPTW** |  | **SMD** | **After IPTW** |  | **SMD** |
| --- | --- | --- | --- | --- | --- | --- |
|  | **DOACs (n=7,896)** | **VKAs (n=6,791)** |  | **DOACs (n=7,893)** | **VKAs (n=6,814)** |  |
| **Antiarrhythmic drugs^*^** |  |  |  |  |  |  |
| Amiodarone | 1,776 (22.49) | 1,583 (23.31) |  | 1,661 (21.04) | 1,708 (25.07) |  |
| Diltiazem | 3,235 (40.97) | 2,839 (41.81) |  | 3,326 (42.13) | 2,739 (40.20) |  |
| Dronedarone | 54 (0.68) | 66 (0.97) |  | 55 (0.69) | 63 (0.93) |  |
| Flecainide | 1,039 (13.16) | 541 (7.97) |  | 1,024 (12.98) | 554 (8.12) |  |
| Propafenone | 28 (0.35) | 23 (0.34) |  | 29 (0.37) | 24 (0.36) |  |
| Sotalol | 823 (10.42) | 898 (13.22) |  | 834 (10.56) | 899 (13.20) |  |
| Verapamil | 844 (10.69) | 754 (11.10) |  | 863 (10.93) | 738 (10.83) |  |
| Multiple | 97 (1.23) | 87 (1.28) |  | 102 (1.30) | 88 (1.29) |  |
| **Demographic characteristics** |  |  |  |  |  |  |
| Age in years, mean (standard deviation) | 70.83 (12.00) | 71.59 (10.83) | -0.066 | 71.22 (11.47) | 71.32 (11.61) | -0.009 |
| Female sex | 3,761 (47.63) | 3,273 (48.20) | -0.011 | 3,783 (47.93) | 3,282 (48.16) | -0.005 |
| Index of Multiple Deprivation |  |  |  |  |  |  |
| 1 | 1,174 (14.87) | 923 (13.59) | 0.037 | 1,124 (14.24) | 981 (14.40) | -0.005 |
| 2 | 1,078 (13.65) | 886 (13.05) | 0.018 | 1,050 (13.31) | 908 (13.32) | <0.001 |
| 3 | 1,076 (13.63) | 937 (13.80) | -0.005 | 1,082 (13.71) | 932 (13.68) | 0.001 |
| 4 | 1,181 (14.96) | 986 (14.52) | 0.012 | 1,163 (14.74) | 1,004 (14.73) | <0.001 |
| 5 | 994 (12.59) | 851 (12.53) | 0.002 | 992 (12.56) | 858 (12.59) | -0.001 |
| Unknown | 2,393 (30.31) | 2,208 (32.51) | -0.047 | 2,482 (31.44) | 2131 (31.27) | 0.004 |
| **Lifestyle variables and clinical measures** |  |  |  |  |  |  |
| Smoking Status |  |  |  |  |  |  |
| Current | 723 (9.16) | 691 (10.18) | -0.035 | 761 (9.64) | 656 (9.62) | 0.001 |
| Former | 3,220 (40.78) | 2,899 (42.69) | -0.039 | 3,296 (41.76) | 2,839 (41.67) | 0.002 |
| Never | 3,932 (49.80) | 3,194 (47.03) | 0.055 | 3,821 (48.41) | 3,305 (48.50) | -0.002 |
| Unknown | 21 (0.27) | 7 (0.10) | 0.040 | 15 (0.19) | 14 (0.21) | -0.004 |
| Body mass index in kg/m^2^ |  |  |  |  |  |  |
| <25 | 1,882 (23.83) | 1,572 (23.15) | 0.016 | 1,853 (23.47) | 1,629 (23.90) | -0.010 |
| 25-29 | 2,738 (34.68) | 2,349 (34.59) | 0.002 | 2,736 (34.67) | 2,346 (34.42) | 0.005 |
| ≥30 | 3,045 (38.56) | 2,660 (39.17) | -0.013 | 3,070 (38.89) | 2,640 (38.75) | 0.003 |
| Unknown | 231 (2.93) | 210 (3.09) | -0.009 | 234 (2.97) | 199 (2.92) | 0.003 |
| Blood pressure control^**^ |  |  |  |  |  |  |
| Normal | 2,319 (29.37) | 1,990 (29.30) | 0.002 | 2,320 (29.40) | 1,995 (29.28) | 0.003 |
| High | 5,565 (70.48) | 4,794 (70.59) | -0.002 | 5,563 (70.47) | 4,811 (70.60) | -0.003 |
| Unknown | 12 (0.15) | 7 (0.10) | 0.014 | 10 (0.13) | 8 (0.12) | 0.003 |
| **Comorbidities** |  |  |  |  |  |  |
| Alcohol-related disorders | 2,375 (30.08) | 1,748 (25.74) | 0.097 | 2,212 (28.03) | 1,906 (27.98) | 0.001 |
| Arterial hypertension | 5,576 (70.62) | 4,896 (72.10) | -0.033 | 5,643 (71.49) | 4,872 (71.49) | <0.001 |
| Ischemic stroke/TIA | 1,045 (13.23) | 855 (12.59) | 0.019 | 1,023 (12.96) | 873 (12.81) | 0.004 |
| Congestive heart failure | 1,818 (23.02) | 1,565 (23.05) | -0.001 | 1,821 (23.07) | 1,557 (22.86) | 0.005 |
| Coronary artery disease | 2,888 (36.58) | 2,662 (39.20) | -0.054 | 3,003 (38.04) | 2,611 (38.31) | -0.006 |
| Peripheral vascular disease | 731 (9.26) | 658 (9.69) | -0.015 | 751 (9.51) | 648 (9,51) | <0.001 |
| Major bleeding | 236 (2.99) | 214 (3.15) | -0.009 | 241 (3.05) | 208 (3.05) | <0.001 |
| Diabetes mellitus | 2,106 (26.67) | 1,717 (25.28) | 0.032 | 2,061 (26.11) | 1,767 (25.93) | 0.004 |
| Liver disease | 341 (4.32) | 237 (3.49) | 0.043 | 311 (3.94) | 267 (3.91) | 0.002 |
| Renal disease^***^ | 3,322 (42.07) | 2,966 (43.68) | -0.033 | 3,388 (42.93) | 2,933 (43.04) | -0.002 |
| Cancer | 418 (5.29) | 327 (4.82) | 0.021 | 396 (5.02) | 341 (5.00) | 0.001 |
| **Comedications** |  |  |  |  |  |  |
| Antiplatelet agents | 3,669 (46.47) | 4,188 (61.67) | -0.309 | 4,219 (53.45) | 3,629 (53.26) | 0.004 |
| Non-steroidal anti-inflammatory drugs | 1,001 (12.68) | 1,048 (15.43) | -0.079 | 1,090 (13.82) | 937 (13.75) | 0.002 |
| Selective serotonin reuptake inhibitors | 802 (10.16) | 656 (9.66) | 0.017 | 777 (9.85) | 698 (10.24) | -0.013 |
| Proton pump inhibitors | 3,703 (46.90) | 3,170 (46.68) | 0.004 | 3,692 (46.77) | 3,195 (46.89) | -0.002 |
| H_2_ blockers | 379 (4.80) | 350 (5.15) | -0.016 | 389 (4.92) | 334 (4.91) | <0.001 |
| N Hospitalizations in the past year |  |  |  |  |  |  |
| 0 | 5,146 (65.17) | 4,351 (64.07) | 0.023 | 5,102 (64.63) | 4,379 (64.26) | 0.008 |
| 1 | 1,832 (23.20) | 1,528 (22.50) | 0.017 | 1,811 (22.95) | 1,589 (23.32) | -0.009 |
| ≥2 | 918 (11.63) | 912 (13.43) | -0.054 | 980 (12.42) | 846 (12.42) | <0.001 |

All values are n (%) unless indicated otherwise.

^*^ Not included in the propensity scores.

^**^ Normal blood pressure control was defined as systolic blood pressure <130 mmHg and diastolic blood pressure <80 mmHg, while high blood pressure was defined as systolic blood pressure ≥130 mmHg or diastolic blood pressure ≥80 mmHg.

^***^ Median values of estimated glomerular filtration rates in the two exposure groups were 69 ml/min per 1.73 m^2^ for DOACs versus 67 ml/min per 1.73 m^2^ for VKAs (both before and after IPTW).

Abbreviations: CPRD, Clinical Practice Research Datalink; IPTW, inverse probability treatment weighting; SMD, standardized mean difference; DOACs, direct oral anticoagulants; VKAs, vitamin K antagonists; TIA, transient ischemic attack.

# **eTable 3. Baseline characteristics of patients in the RAMQ**

|  | **Before IPTW** |  | **SMD** | **After IPTW** |  | **SMD** |
| --- | --- | --- | --- | --- | --- | --- |
|  | **DOACs (n=21,175)** | **VKAs (8,573)** |  | **DOACs (n=21,173)** | **VKAs (n=8,587)** |  |
| **Antiarrhythmic drugs^*^** |  |  |  |  |  |  |
| Amiodarone | 5,512 (26.03) | 3,258 (38.00) |  | 5,987 (28.28) | 2,675 (31.15) |  |
| Diltiazem | 9,758 (46,08) | 3,909 (45.60) |  | 9,654 (45.60) | 4,214 (49.07) |  |
| Flecainide | 1,330 (6.28) | 139 (1.62) |  | 1,195 (5.64) | 198 (2.31) |  |
| Propafenone | 860 (4.06) | 210 (2.45) |  | 778 (3.67) | 281 (3.27) |  |
| Sotalol | 2,366 (11.17) | 502 (5.86) |  | 2,235 (10.56) | 605 (7.05) |  |
| Verapamil | 569 (2.69) | 280 (3.27) |  | 566 (2.67) | 315 (3.67) |  |
| Multiple | 780 (3.68) | 275 (3.21) |  | 758 (3.58) | 299 (3.58) |  |
| **Demographic characteristics** |  |  |  |  |  |  |
| Age in years, mean (standard deviation) | 74.92 (9.97) | 76.82 (9.45) | -0.196 | 75.36 (9.91) | 76.12 (9.79) | -0.077 |
| Female sex | 11,479 (54.21) | 4,307 (50.24) | 0.080 | 11,251 (53.14) | 4,598 (53.55) | -0.008 |
| **Comorbidities** |  |  |  |  |  |  |
| Alcohol-related disorders | 1,082 (5.11) | 444 (5.18) |  | 1,084 (5.12) | 437 (5.09) | 0.001 |
| Arterial hypertension | 17,958 (84.91) | 7,850 (91.57) | -0.211 | 18,369 (86.76) | 7,463 (86.91) | -0.004 |
| Ischemic stroke/TIA | 1,496 (7.06) | 738 (8.61) | -0.058 | 1,599 (7.55) | 657 (7.65) | -0.004 |
| Congestive heart failure | 5,426 (25.62) | 3,245 (37.85) | -0.265 | 6,174 (29.16) | 2,514 (29.28) | -0.003 |
| Coronary artery disease | 9,582 (45.25) | 5,423 (63.26) | -0.368 | 10,664 (50.17) | 4,274 (49.78) | 0.012 |
| Peripheral vascular disease | 2,605 (12.30) | 1,680 (19.60) | -0.200 | 3,036 (14.34) | 1,219 (14.19) | 0.004 |
| Major bleeding | 2,412 (11.39) | 1,450 (16.91) | -0.159 | 2,757 (13.02) | 1,115 (12.99) | 0.001 |
| Diabetes mellitus | 6,146 (29.02) | 3,372 (39.33) | -0.219 | 6,757 (31.91) | 2,729 (31.78) | 0.003 |
| Liver disease | 1,215 (5.74) | 585 (6.82) | -0.045 | 1,275 (6.02) | 514 (5.99) | 0.001 |
| Renal disease | 3,014 (14.23) | 2,625 (30.62) | -0.401 | 4,014 (18.96) | 1,625 (18.92) | 0.001 |
| Cancer | 3,819 (18.04) | 1,512 (17.64) | 0.010 | 3,800 (17.95) | 1,558 (18.15) | -0.005 |
| **Comedications** |  |  |  |  |  |  |
| Antiplatelet agents | 11,688 (55.20) | 5,809 (67.76) | -0.260 | 12,466 (58.88) | 5,078 (59.14) | -0.005 |
| Non-steroidal anti-inflammatory drugs | 3,628 (17.13) | 1,368 (15.96) | 0.031 | 3,546 (16.75) | 1,438 (16.75) | <0.001 |
| Selective serotonin reuptake inhibitors | 2,252 (10.64) | 863 (10.07) | 0.019 | 2,223 (10.50) | 920 (10.71) | -0.007 |
| Proton pump inhibitors | 10,308 (48.68) | 4,360 (50.86) | -0.044 | 10,443 (49.32) | 4,271 (49.74) | -0.008 |
| H_2_ blockers | 494 (2.33) | 163 (1.90) | 0.030 | 468 (2.21) | 194 (2.26) | -0.003 |
| N Non-anticoagulant drugs in the past year |  |  |  |  |  |  |
| 0-7 | 6,851 (32.35) | 2,134 (24.89) | 0.166 | 6,373 (30.10) | 2,517 (29.32) | 0.017 |
| 8-13 | 7,442 (35.15) | 2,980 (34.76) | 0.008 | 7,434 (35.11) | 3,052 (35.54) | -0.009 |
| ≥14 | 6,882 (32.50) | 3,459 (40.35) | -0.164 | 7,366 (34.79) | 3,018 (35.15) | -0.008 |
| N Hospitalizations in the past year |  |  |  |  |  |  |
| 0 | 6,825 (32.23) | 1,308 (15.26) | 0.407 | 5,792 (27.35) | 2,364 (27.53) | -0.004 |
| 1 | 10,192 (48.13) | 4,344 (50.67) | -0.051 | 10,346 (48.87) | 4,193 (48.83) | 0.001 |
| ≥2 | 4,158 (19.64) | 2,921 (34.07) | -0.330 | 5,035 (23.78) | 2,030 (23.64) | 0.003 |

All values are n (%) unless indicated otherwise.

^*^ Not included in the propensity scores.

Abbreviations: RAMQ, Régie de l'Assurance-Maladie du Québec; IPTW, inverse probability treatment weighting; SMD, standardized mean difference; DOACs, direct oral anticoagulants; VKAs, vitamin K antagonists; TIA, transient ischemic attack.

# **eTable 4. Risk of ischemic stroke associated with use of DOACs compared with use of VKAs among patients with NVAF treated with antiarrhythmics (stratification by demographics)**

|  | **N**  **Patients** | **N**  **Events** | **N**  **PY** | | **IR^*^** | **Crude HR**  **(95% CI)** | **IPTW HR**  **(95% CI)** | **Pooled HR**  **(95% CI)** | **I^2^** | |
| --- | --- | --- | --- | --- | --- | --- | --- | --- | --- | --- |
| **<70 years** |  |  |  |  | |  |  | 0.91 (0.60-1.39) | 67% |  |
| **CPRD** |  |  |  |  | |  |  |  |  |  |
| DOACs | 3,299 | 14 | 2,637 | 5.31 | | 1.73 (0.72-4.16) | 1.70 (0.75-3.86) |  |  |  |
| VKAs | 2,669 | 8 | 2,835 | 2.82 | | 1.00 (reference) | 1.00 (reference) |  |  |  |
| **RAMQ** |  |  |  |  | |  |  |  |  |  |
| DOACs | 5,877 | 66 | 9,271 | 7.12 | | 0.54 (0.34-0.87) | 0.73 (0.45-1.19) |  |  |  |
| VKAs | 1,825 | 24 | 1,559 | 15.40 | | 1.00 (reference) | 1.00 (reference) |  |  |  |
| **≥70 years** |  |  |  |  | |  |  | 0.86 (0.71-1.04) | 0% |  |
| **CPRD** |  |  |  |  | |  |  |  |  |  |
| DOACs | 4,597 | 35 | 3,732 | 9.38 | | 0.82 (0.53-1.27) | 0.82 (0.53-1.27) |  |  |  |
| VKAs | 4,122 | 46 | 4,409 | 10.43 | | 1.00 (reference) | 1.00 (reference) |  |  |  |
| **RAMQ** |  |  |  |  | |  |  |  |  |  |
| DOACs | 15,298 | 311 | 23,137 | 13.44 | | 0.76 (0.62-0.94) | 0.89 (0.72-1.09) |  |  |  |
| VKAs | 6,748 | 131 | 6,645 | 19.72 | | 1.00 (reference) | 1.00 (reference) |  |  |  |
|  |  |  |  |  | |  |  |  |  |  |
| **Female sex** |  |  |  |  | |  |  | 0.89 (0.70-1.12) | 11% |  |
| **CPRD** |  |  |  |  | |  |  |  |  |  |
| DOACs | 3,761 | 26 | 3,239 | 8.03 | | 1.30 (0.72-2.33) | 1.19 (0.66-2.13) |  |  |  |
| VKAs | 3,273 | 20 | 3,689 | 5.42 | | 1.00 (reference) | 1.00 (reference) |  |  |  |
| **RAMQ** |  |  |  |  | |  |  |  |  |  |
| DOACs | 11,479 | 212 | 18,748 | 11.31 | | 0.69 (0.54-0.89) | 0.84 (0.65-1.09) |  |  |  |
| VKAs | 4,307 | 87 | 4,728 | 18.40 | | 1.00 (reference) | 1.00 (reference) |  |  |  |
| **Male sex** |  |  |  |  | |  |  | 0.81 (0.63-1.04) | 0% |  |
| **CPRD** |  |  |  |  | |  |  |  |  |  |
| DOACs | 4,135 | 23 | 3,130 | 7.35 | | 0.72 (0.42-1.23) | 0.72 (0.43-1.20) |  |  |  |
| VKAs | 3,518 | 34 | 3,555 | 9.57 | | 1.00 (reference) | 1.00 (reference) |  |  |  |
| **RAMQ** |  |  |  |  | |  |  |  |  |  |
| DOACs | 9,696 | 165 | 13,660 | 12.08 | | 0.69 (0.52-0.92) | 0.84 (0.63-1.12) |  |  |  |
| VKAs | 4,266 | 68 | 3,475 | 19.57 | | 1.00 (reference) | 1.00 (reference) |  |  |  |

^*^ IR per 1,000 PY.

Abbreviations: DOACs, direct oral anticoagulants; VKAs, vitamin K antagonists; NVAF, non-valvular atrial fibrillation; PY, patient years; IR, incidence rate; HR, hazard ratio; CI, confidence interval. IPTW, inverse probability of treatment weighting; CPRD, Clinical Practice Research Datalink; RAMQ, Régie de l'Assurance-Maladie du Québec.

# **eTable 5. Risk of ischemic stroke associated with use of DOACs compared with use of VKAs among patients with NVAF treated with antiarrhythmics (stratification by baseline risk and individual DOACs)**

|  | **N**  **Patients** | **N**  **Events** | **N**  **PY** | **IR^*^** | **Crude HR**  **(95% CI)** | **IPTW HR**  **(95% CI)** | **Pooled HR**  **(95% CI)** | | **I^2^** | |  |
| --- | --- | --- | --- | --- | --- | --- | --- | --- | --- | --- | --- |
| **CHA_2_DS_2_-VASC 0-3** |  |  |  |  |  |  | | 0.90 (0.59-1.37) | | 0% | |
| **CPRD** |  |  |  |  |  |  | |  | |  | |
| DOACs | 3,951 | 12 | 3,178 | 3.78 | 0.85 (0.40-1.84) | 0.95 (0.47-1.92) | |  | |  | |
| VKAs | 3,291 | 15 | 3,509 | 4.27 | 1.00 (reference) | 1.00 (reference) | |  | |  | |
| **RAMQ** |  |  |  |  |  |  | |  | |  | |
| DOACs | 7,997 | 87 | 13,182 | 6.60 | 0.75 (0.45-1.25) | 0.87 (0.52-1.48) | |  | |  | |
| VKAs | 1,875 | 18 | 1,835 | 9.81 | 1.00 (reference) | 1.00 (reference) | |  | |  | |
| **CHA_2_DS_2_-VASC ≥4** |  |  |  |  |  |  | | 0.86 (0.71-1.03) | | 0% | |
| **CPRD** |  |  |  |  |  |  | |  | |  | |
| DOACs | 3,945 | 37 | 3,191 | 11.59 | 0.99 (0.63-1.56) | 0.90 (0.57-1.42) | |  | |  | |
| VKAs | 3,500 | 39 | 3,734 | 10.44 | 1.00 (reference) | 1.00 (reference) | |  | |  | |
| **RAMQ** |  |  |  |  |  |  | |  | |  | |
| DOACs | 13,178 | 290 | 19,226 | 15.08 | 0.79 (0.64-0.96) | 0.85 (0.69-1.04) | |  | |  | |
| VKAs | 6,698 | 137 | 6,369 | 21.51 | 1.00 (reference) | 1.00 (reference) | |  | |  | |
|  |  |  |  |  |  |  | |  | |  | |
| **Apixaban** |  |  |  |  |  |  | | 0.67 (0.53-0.86) | | 0% | |
| **CPRD** |  |  |  |  |  |  | |  | |  | |
| Apixaban | 3,573 | 21 | 2,564 | 8.19 | 0.88 (0.50-1.53) | 0.78 (0.45-1.36) | |  | |  | |
| VKAs | 3,797 | 30 | 3,682 | 8.15 | 1.00 (reference) | 1.00 (reference) | |  | |  | |
| **RAMQ** |  |  |  |  |  |  | |  | |  | |
| Apixaban | 11,229 | 196 | 14,855 | 13.19 | 0.64 (0.48-0.85) | 0.65 (0.50-0.86) | |  | |  | |
| VKAs | 3,483 | 63 | 2,531 | 24.89 | 1.00 (reference) | 1.00 (reference) | |  | |  | |
| **Rivaroxaban** |  |  |  |  |  |  | | 0.94 (0.76-1.16) | | 0% | |
| **CPRD** |  |  |  |  |  |  | |  | |  | |
| Rivaroxaban | 3,310 | 21 | 2,791 | 7.52 | 1.05 (0.61-1.81) | 1.12 (0.66-1.90) | |  | |  | |
| VKAs | 5,164 | 36 | 5,325 | 6.76 | 1.00 (reference) | 1.00 (reference) | |  | |  | |
| **RAMQ** |  |  |  |  |  |  | |  | |  | |
| Rivaroxaban | 7,204 | 119 | 11,127 | 10.70 | 0.63 (0.50-0.80) | 0.91 (0.72-1.15) | |  | |  | |
| VKAs | 8,407 | 154 | 7,995 | 19.26 | 1.00 (reference) | 1.00 (reference) | |  | |  | |

^*^ IR per 1,000 PY.

Abbreviations: DOACs, direct oral anticoagulants; VKAs, vitamin K antagonists; NVAF, non-valvular atrial fibrillation; CHA_2_DS_2_-VASc, congestive heart failure, hypertension, age ≥75 years, diabetes mellitus, stroke, vascular disease, age 65-74 years, sex; PY, patient years; IR, incidence rate; HR, hazard ratio; CI, confidence interval. IPTW, inverse probability of treatment weighting; CPRD, Clinical Practice Research Datalink; RAMQ, Régie de l'Assurance-Maladie du Québec.

# **eTable 6. Risk of ischemic stroke associated with use of DOACs compared with use of VKAs among patients with NVAF treated with antiarrhythmics (stratification by types of antiarrhythmics)**

|  | **N**  **Patients** | **N**  **Events** | **N**  **PY** | **IR^*^** | **Crude HR**  **(95% CI)** | **IPTW HR**  **(95% CI)** | **Pooled HR**  **(95% CI)** | **I^2^** |
| --- | --- | --- | --- | --- | --- | --- | --- | --- |
| **Prevalent antiarrhythmics use** |  |  |  |  |  |  | 0.88 (0.69-1.14) | 31% |
| **CPRD** |  |  |  |  |  |  |  |  |
| DOACs | 4,911 | 33 | 3,957 | 8.34 | 1.18 (0.74-1.90) | 1.13 (0.70-1.81) |  |  |
| VKAs | 5,240 | 37 | 5,696 | 6.50 | 1.00 (reference) | 1.00 (reference) |  |  |
| **RAMQ** |  |  |  |  |  |  |  |  |
| DOACs | 7,686 | 149 | 11,485 | 12.97 | 0.68 (0.50-0.92) | 0.80 (0.59-1.08) |  |  |
| VKAs | 3,122 | 62 | 2,908 | 21.32 | 1.00 (reference) | 1.00 (reference) |  |  |
| **New antiarrhythmics use** |  |  |  |  |  |  | 0.84 (0.66-1.06) | 24% |
| **CPRD** |  |  |  |  |  |  |  |  |
| DOACs | 2,985 | 16 | 2,412 | 6.63 | 0.55 (0.28-1.09) | 0.58 (0.29-1.14) |  |  |
| VKAs | 1,551 | 17 | 1,548 | 10.99 | 1.00 (reference) | 1.00 (reference) |  |  |
| **RAMQ** |  |  |  |  |  |  |  |  |
| DOACs | 13,489 | 228 | 20,923 | 10.90 | 0.71 (0.56-0.90) | 0.71 (0.56-0.90) |  |  |
| VKAs | 5,451 | 93 | 5,295 | 17.56 | 1.00 (reference) | 1.00 (reference) |  |  |
|  |  |  |  |  |  |  |  |  |
| **Rate control** |  |  |  |  |  |  | 0.86 (0.68-1.08) | 52% |
| **CPRD** |  |  |  |  |  |  |  |  |
| DOACs | 4,080 | 31 | 3,213 | 9.65 | 1.34 (0.79-2.26) | 1.21 (0.72-2.02) |  |  |
| VKAs | 3,594 | 27 | 3,990 | 6.77 | 1.00 (reference) | 1.00 (reference) |  |  |
| **RAMQ** |  |  |  |  |  |  |  |  |
| DOACs | 12,058 | 214 | 17,222 | 12.43 | 0.66 (0.52-0.84) | 0.79 (0.61-1.02) |  |  |
| VKAs | 4,418 | 93 | 4,610 | 20.17 | 1.00 (reference) | 1.00 (reference) |  |  |
| **Rhythm control** |  |  |  |  |  |  | 0.90 (0.67-1.21) | 0% |
| **CPRD** |  |  |  |  |  |  |  |  |
| DOACs | 3,722 | 16 | 2,830 | 5.65 | 0.71 (0.37-1.38) | 0.71 (0.37-1.34) |  |  |
| VKAs | 3,116 | 20 | 2,757 | 7.25 | 1.00 (reference) | 1.00 (reference) |  |  |
| **RAMQ** |  |  |  |  |  |  |  |  |
| DOACs | 8,788 | 127 | 11,346 | 11.19 | 0.72 (0.53-1.00) | 0.96 (0.69-1.34) |  |  |
| VKAs | 3,999 | 55 | 2,949 | 18.65 | 1.00 (reference) | 1.00 (reference) |  |  |
|  |  |  |  |  |  |  |  |  |
| **No/weak CYP3A4 inhibitors** |  |  |  |  |  |  | 0.90 (0.66-1.24) | 29% |
| **CPRD** |  |  |  |  |  |  |  |  |
| DOACs | 1,892 | 5 | 1,541 | 3.25 | 0.43 (0.15-1.27) | 0.48 (0.16-1.42) |  |  |
| VKAs | 1,465 | 10 | 1,524 | 6.56 | 1.00 (reference) | 1.00 (reference) |  |  |
| **RAMQ** |  |  |  |  |  |  |  |  |
| DOACs | 3,227 | 40 | 4,812 | 8.31 | 0.56 (0.28-1.13) | 0.80 (0.36-1.74) |  |  |
| VKAs | 712 | 10 | 582 | 17.17 | 1.00 (reference) | 1.00 (reference) |  |  |
| **Moderate CYP3A4 inhibitors** |  |  |  |  |  |  | 0.84 (0.68-1.04) | 0% |
| **CPRD** |  |  |  |  |  |  |  |  |
| DOACs | 5,944 | 44 | 4,592 | 9.58 | 1.11 (0.73-1.70) | 1.01 (0.66-1.53) |  |  |
| VKAs | 5,284 | 43 | 5,441 | 7.90 | 1.00 (reference) | 1.00 (reference) |  |  |
| **RAMQ** |  |  |  |  |  |  |  |  |
| DOACs | 17,789 | 321 | 25,085 | 12.80 | 0.72 (0.59-0.88) | 0.85 (0.69-1.03) |  |  |
| VKAs | 7,820 | 145 | 7,388 | 19.63 | 1.00 (reference) | 1.00 (reference) |  |  |

^*^ IR per 1,000 PY.

Abbreviations: DOACs, direct oral anticoagulants; VKAs, vitamin K antagonists; NVAF, non-valvular atrial fibrillation; PY, patient years; IR, incidence rate; HR, hazard ratio; CI, confidence interval. IPTW, inverse probability of treatment weighting; CPRD, Clinical Practice Research Datalink; RAMQ, Régie de l'Assurance-Maladie du Québec; CYP3A4, cytochrome P450 3A4.

# **eTable 7. Risk of major bleeding associated with use of DOACs compared with use of VKAs among patients with NVAF treated with antiarrhythmics (stratification by demographics)**

|  | **N**  **Patients** | **N**  **Events** | **N**  **PY** | **IR^*^** | **Crude HR**  **(95% CI)** | **IPTW HR**  **(95% CI)** | **Pooled HR**  **(95% CI)** | **I^2^** |
| --- | --- | --- | --- | --- | --- | --- | --- | --- |
| **<70 years** |  |  |  |  |  |  | 0.69 (0.58-0.82) | 57% |
| **CPRD** |  |  |  |  |  |  |  |  |
| DOACs | 3,299 | 76 | 2,594 | 29.30 | 0.87 (0.64-1.19) | 0.84 (0.62-1.15) |  |  |
| VKAs | 2,669 | 85 | 2,736 | 31.07 | 1.00 (reference) | 1.00 (reference) |  |  |
| **RAMQ** |  |  |  |  |  |  |  |  |
| DOACs | 5,877 | 310 | 9,022 | 34.36 | 0.52 (0.42-0.64) | 0.63 (0.50-0.78) |  |  |
| VKAs | 1,825 | 119 | 1,486 | 80.09 | 1.00 (reference) | 1.00 (reference) |  |  |
| **≥70 years** |  |  |  |  |  |  | 0.90 (0.82-0.98) | 0% |
| **CPRD** |  |  |  |  |  |  |  |  |
| DOACs | 4,597 | 160 | 3,656 | 43.77 | 1.04 (0.83-1.29) | 0.92 (0.74-1.15) |  |  |
| VKAs | 4,122 | 171 | 4,310 | 39.68 | 1.00 (reference) | 1.00 (reference) |  |  |
| **RAMQ** |  |  |  |  |  |  |  |  |
| DOACs | 15,298 | 1,259 | 22,371 | 56.28 | 0.74 (0.67-0.82) | 0.89 (0.80-0.99) |  |  |
| VKAs | 6,748 | 545 | 6,395 | 85.22 | 1.00 (reference) | 1.00 (reference) |  |  |
|  |  |  |  |  |  |  |  |  |
| **Female sex** |  |  |  |  |  |  | 0.84 (0.74-0.94) | 0% |
| **CPRD** |  |  |  |  |  |  |  |  |
| DOACs | 3,761 | 112 | 3,167 | 35.36 | 1.01 (0.78-1.31) | 0.85 (0.66-1.11) |  |  |
| VKAs | 3,273 | 116 | 3,578 | 32.42 | 1.00 (reference) | 1.00 (reference) |  |  |
| **RAMQ** |  |  |  |  |  |  |  |  |
| DOACs | 11,479 | 809 | 18,150 | 44.57 | 0.70 (0.62-0.80) | 0.83 (0.73-0.95) |  |  |
| VKAs | 4,307 | 319 | 4,578 | 69.68 | 1.00 (reference) | 1.00 (reference) |  |  |
| **Male sex** |  |  |  |  |  |  | 0.84 (0.75-0.94) | 0% |
| **CPRD** |  |  |  |  |  |  |  |  |
| DOACs | 4,135 | 124 | 3,082 | 40.23 | 0.94 (0.73-1.20) | 0.93 (0.73-1.19) |  |  |
| VKAs | 3,518 | 140 | 3,467 | 40.38 | 1.00 (reference) | 1.00 (reference) |  |  |
| **RAMQ** |  |  |  |  |  |  |  |  |
| DOACs | 9,696 | 760 | 13,243 | 57.39 | 0.65 (0.57-0.74) | 0.82 (0.72-0.93) |  |  |
| VKAs | 4,266 | 345 | 3,302 | 104.47 | 1.00 (reference) | 1.00 (reference) |  |  |

^*^ IR per 1,000 PY.

Abbreviations: DOACs, direct oral anticoagulants; VKAs, vitamin K antagonists; NVAF, non-valvular atrial fibrillation; PY, patient years; IR, incidence rate; HR, hazard ratio; CI, confidence interval. IPTW, inverse probability of treatment weighting; CPRD, Clinical Practice Research Datalink; RAMQ, Régie de l'Assurance-Maladie du Québec.

# **eTable 8. Risk of major bleeding associated with use of DOACs compared with use of VKAs among patients with NVAF treated with antiarrhythmics (stratification by baseline risk and individual DOACs)**

|  | **N**  **Patients** | **N**  **Events** | **N**  **PY** | **IR^*^** | **Crude HR**  **(95% CI)** | **IPTW HR**  **(95% CI)** | **Pooled HR**  **(95% CI)** | **I^2^** |
| --- | --- | --- | --- | --- | --- | --- | --- | --- |
| **HAS-BLED 0-3^**^** |  |  |  |  |  |  | 0.82 (0.74-0.90) | 0% |
| **CPRD** |  |  |  |  |  |  |  |  |
| DOACs | 5,278 | 133 | 4,139 | 32.14 | 0.90 (0.71-1.14) | 0.87 (0.69-1.10) |  |  |
| VKAs | 4,292 | 150 | 4,439 | 33.79 | 1.00 (reference) | 1.00 (reference) |  |  |
| **RAMQ** |  |  |  |  |  |  |  |  |
| DOACs | 18,660 | 1,281 | 28,466 | 45.00 | 0.71 (0.63-0.79) | 0.81 (0.72-0.90) |  |  |
| VKAs | 6,513 | 438 | 6,077 | 72.07 | 1.00 (reference) | 1.00 (reference) |  |  |
| **HAS-BLED ≥4^**^** |  |  |  |  |  |  | 0.94 (0.81-1.09) | 0% |
| **CPRD** |  |  |  |  |  |  |  |  |
| DOACs | 2,618 | 103 | 2,111 | 48.79 | 1.11 (0.84-1.45) | 0.88 (0.67-1.15) |  |  |
| VKAs | 2,499 | 106 | 2,606 | 40.67 | 1.00 (reference) | 1.00 (reference) |  |  |
| **RAMQ** |  |  |  |  |  |  |  |  |
| DOACs | 2,515 | 288 | 2,928 | 98.38 | 0.85 (0.71-1.01) | 0.97 (0.81-1.16) |  |  |
| VKAs | 2,060 | 226 | 1,803 | 125.33 | 1.00 (reference) | 1.00 (reference) |  |  |
|  |  |  |  |  |  |  |  |  |
| **Apixaban** |  |  |  |  |  |  | 0.71 (0.63-0.81) | 74% |
| **CPRD** |  |  |  |  |  |  |  |  |
| Apixaban | 3,573 | 92 | 2,533 | 36.32 | 0.97 (0.74-1.27) | 0.91 (0.69-1.19) |  |  |
| VKAs | 3,797 | 124 | 3,600 | 34.44 | 1.00 (reference) | 1.00 (reference) |  |  |
| **RAMQ** |  |  |  |  |  |  |  |  |
| Apixaban | 11,229 | 736 | 14,453 | 50.93 | 0.54 (0.47-0.63) | 0.67 (0.58-0.77) |  |  |
| VKAs | 3,483 | 273 | 2,423 | 112.69 | 1.00 (reference) | 1.00 (reference) |  |  |
| **Rivaroxaban** |  |  |  |  |  |  | 1.02 (0.92-1.13) | 0% |
| **CPRD** |  |  |  |  |  |  |  |  |
| Rivaroxaban | 3,310 | 108 | 2,732 | 39.53 | 1.01 (0.79-1.27) | 0.96 (0.76-1.22) |  |  |
| VKAs | 5,164 | 193 | 5,183 | 37.24 | 1.00 (reference) | 1.00 (reference) |  |  |
| **RAMQ** |  |  |  |  |  |  |  |  |
| Rivaroxaban | 7,204 | 578 | 10,735 | 53.84 | 0.72 (0.64-0.81) | 1.04 (0.93-1.16) |  |  |
| VKAs | 8,407 | 653 | 7,677 | 85.06 | 1.00 (reference) | 1.00 (reference) |  |  |

^*^ IR per 1,000 PY.

^**^ HAS-BLED did not include labile international normalized ratio due to lack of standardized reporting.

Abbreviations: DOACs, direct oral anticoagulants; VKAs, vitamin K antagonists; NVAF, non-valvular atrial fibrillation; PY, patient years; IR, incidence rate; HR, hazard ratio; CI, confidence interval. IPTW, inverse probability of treatment weighting; CPRD, Clinical Practice Research Datalink; RAMQ, Régie de l'Assurance-Maladie du Québec; HAS-BLED, hypertension, abnormal renal or liver function, stroke, bleeding, elderly, drugs or excess alcohol use.

# **eTable 9. Risk of major bleeding associated with use of DOACs compared with use of VKAs among patients with NVAF treated with antiarrhythmics (stratification by types of antiarrhythmics)**

|  | **N**  **Patients** | **N**  **Events** | **N**  **PY** | **IR^*^** | **Crude HR**  **(95% CI)** | **IPTW HR**  **(95% CI)** | **Pooled HR**  **(95% CI)** | **I^2^** |
| --- | --- | --- | --- | --- | --- | --- | --- | --- |
| **Prevalent antiarrhythmics use** |  |  |  |  |  |  | 0.77 (0.68-0.88) | 0% |
| **CPRD** |  |  |  |  |  |  |  |  |
| DOACs | 4,911 | 135 | 3,903 | 34.59 | 0.86 (0.69-1.07) | 0.78 (0.63-0.97) |  |  |
| VKAs | 5,240 | 208 | 5,543 | 37.52 | 1.00 (reference) | 1.00 (reference) |  |  |
| **RAMQ** |  |  |  |  |  |  |  |  |
| DOACs | 7,686 | 544 | 11,120 | 48.92 | 0.62 (0.53-0.72) | 0.77 (0.66-0.90) |  |  |
| VKAs | 3,122 | 247 | 2,783 | 88.76 | 1.00 (reference) | 1.00 (reference) |  |  |
| **New antiarrhythmics use** |  |  |  |  |  |  | 0.90 (0.80-1.00) | 75% |
| **CPRD** |  |  |  |  |  |  |  |  |
| DOACs | 2,985 | 101 | 2,347 | 43.03 | 1.29 (0.91-1.83) | 1.24 (0.89-1.75) |  |  |
| VKAs | 1,551 | 48 | 1,502 | 31.96 | 1.00 (reference) | 1.00 (reference) |  |  |
| **RAMQ** |  |  |  |  |  |  |  |  |
| DOACs | 13,489 | 1,025 | 20,273 | 50.56 | 0.71 (0.63-0.79) | 0.86 (0.77-0.97) |  |  |
| VKAs | 5,451 | 417 | 5,098 | 81.80 | 1.00 (reference) | 1.00 (reference) |  |  |
|  |  |  |  |  |  |  |  |  |
| **Rate control** |  |  |  |  |  |  | 0.88 (0.78-0.99) | 20% |
| **CPRD** |  |  |  |  |  |  |  |  |
| DOACs | 4,080 | 132 | 3,144 | 41.98 | 1.10 (0.86-1.39) | 0.99 (0.78-1.27) |  |  |
| VKAs | 3,594 | 136 | 3,882 | 35.03 | 1.00 (reference) | 1.00 (reference) |  |  |
| **RAMQ** |  |  |  |  |  |  |  |  |
| DOACs | 12,058 | 811 | 16,720 | 48.50 | 0.69 (0.60-0.78) | 0.85 (0.74-0.97) |  |  |
| VKAs | 4,418 | 339 | 4,454 | 76.10 | 1.00 (reference) | 1.00 (reference) |  |  |
| **Rhythm control** |  |  |  |  |  |  | 0.87 (0.76-0.99) | 0% |
| **CPRD** |  |  |  |  |  |  |  |  |
| DOACs | 3,722 | 92 | 2,780 | 33.09 | 0.84 (0.63-1.12) | 0.83 (0.63-1.11) |  |  |
| VKAs | 3,116 | 102 | 2,686 | 37.97 | 1.00 (reference) | 1.00 (reference) |  |  |
| **RAMQ** |  |  |  |  |  |  |  |  |
| DOACs | 8,788 | 593 | 10,998 | 53.92 | 0.68 (0.59-0.79) | 0.88 (0.75-1.02) |  |  |
| VKAs | 3,999 | 267 | 2,822 | 94.61 | 1.00 (reference) | 1.00 (reference) |  |  |
|  |  |  |  |  |  |  |  |  |
| **No/weak CYP3A4 inhibitors** |  |  |  |  |  |  | 0.86 (0.75-0.99) | 0% |
| **CPRD** |  |  |  |  |  |  |  |  |
| DOACs | 1,892 | 33 | 1,523 | 21.66 | 0.71 (0.45-1.12) | 0.73 (0.46-1.15) |  |  |
| VKAs | 1,465 | 44 | 1,486 | 29.62 | 1.00 (reference) | 1.00 (reference) |  |  |
| **RAMQ** |  |  |  |  |  |  |  |  |
| DOACs | 3,227 | 128 | 4,742 | 26.99 | 0.68 (0.45-1.03) | 0.85 (0.54-1.34) |  |  |
| VKAs | 712 | 27 | 575 | 46.93 | 1.00 (reference) | 1.00 (reference) |  |  |
| **Moderate CYP3A4 inhibitors** |  |  |  |  |  |  | 0.88 (0.79-0.99) | 7% |
| **CPRD** |  |  |  |  |  |  |  |  |
| DOACs | 5,944 | 200 | 4,492 | 44.52 | 1.07 (0.88-1.30) | 0.96 (0.79-1.17) |  |  |
| VKAs | 5,284 | 204 | 5,292 | 38.55 | 1.00 (reference) | 1.00 (reference) |  |  |
| **RAMQ** |  |  |  |  |  |  |  |  |
| DOACs | 17,789 | 1,369 | 24,236 | 56.49 | 0.71 (0.65-0.78) | 0.86 (0.78-0.94) |  |  |
| VKAs | 7,820 | 626 | 7,092 | 88.27 | 1.00 (reference) | 1.00 (reference) |  |  |

^*^ IR per 1,000 PY.

Abbreviations: DOACs, direct oral anticoagulants; VKAs, vitamin K antagonists; NVAF, non-valvular atrial fibrillation; PY, patient years; IR, incidence rate; HR, hazard ratio; CI, confidence interval. IPTW, inverse probability of treatment weighting; CPRD, Clinical Practice Research Datalink; RAMQ, Régie de l'Assurance-Maladie du Québec; CYP3A4, cytochrome P450 3A4.

# **eTable 10. Risk of ischemic stroke associated with use of DOACs compared with use of VKAs among patients with NVAF treated with antiarrhythmics (sensitivity analyses)**

|  | **N**  **Patients** | **N**  **Events** | **N**  **PY** | **IR^*^** | **Crude HR**  **(95% CI)** | **IPTW HR**  **(95% CI)** | **Pooled HR**  **(95% CI)** | **I^2^** |
| --- | --- | --- | --- | --- | --- | --- | --- | --- |
| **15-day grace period** |  |  |  |  |  |  | 0.93 (0.76-1.13) | 0% |
| **CPRD** |  |  |  |  |  |  |  |  |
| DOACs | 7,896 | 41 | 4,222 | 9.71 | 1.00 (0.65-1.55) | 0.98 (0.64-1.50) |  |  |
| VKAs | 6,791 | 41 | 4,398 | 9.32 | 1.00 (reference) | 1.00 (reference) |  |  |
| **RAMQ** |  |  |  |  |  |  |  |  |
| DOACs | 21,175 | 302 | 24,671 | 12.24 | 0.72 (0.58-0.89) | 0.91 (0.73-1.15) |  |  |
| VKAs | 8,573 | 113 | 5,635 | 20.05 | 1.00 (reference) | 1.00 (reference) |  |  |
| **Stricter outcome definition** |  |  |  |  |  |  | 0.84 (0.67-1.05) | 0% |
| **CPRD** |  |  |  |  |  |  |  |  |
| DOACs | 7,896 | 39 | 6,376 | 6.12 | 0.89 (0.58-1.37) | 0.83 (0.54-1.28) |  |  |
| VKAs | 6,791 | 45 | 7,246 | 6.21 | 1.00 (reference) | 1.00 (reference) |  |  |
| **RAMQ** |  |  |  |  |  |  |  |  |
| DOACs | 21,175 | 211 | 32,499 | 6.49 | 0.75 (0.58-0.98) | 0.85 (0.65-1.10) |  |  |
| VKAs | 8,573 | 79 | 8,228 | 9.60 | 1.00 (reference) | 1.00 (reference) |  |  |
| **Inclusion of fatal events** |  |  |  |  |  |  | 0.86 (0.72-1.03) | 0% |
| **CPRD** |  |  |  |  |  |  |  |  |
| DOACs | 7,896 | 51 | 6,369 | 8.01 | 0.95 (0.65-1.40) | 0.91 (0.62-1.33) |  |  |
| VKAs | 6,791 | 56 | 7,243 | 7.73 | 1.00 (reference) | 1.00 (reference) |  |  |
| **RAMQ^**^** |  |  |  |  |  |  |  |  |
| DOACs | 16,103 | 254 | 20,823 | 12.20 | 0.67 (0.55-0.82) | 0.84 (0.69-1.04) |  |  |
| VKAs | 8,213 | 150 | 7,655 | 19.59 | 1.00 (reference) | 1.00 (reference) |  |  |
| **Exclusion of prior events** |  |  |  |  |  |  | 0.86 (0.70-1.05) | 0% |
| **CPRD** |  |  |  |  |  |  |  |  |
| DOACs | 6,851 | 27 | 5,488 | 4.92 | 0.79 (0.48-1.31) | 0.81 (0.49-1.33) |  |  |
| VKAs | 5,936 | 36 | 6,245 | 5.76 | 1.00 (reference) | 1.00 (reference) |  |  |
| **RAMQ** |  |  |  |  |  |  |  |  |
| DOACs | 19,679 | 303 | 30,219 | 10.03 | 0.71 (0.57-0.88) | 0.87 (0.70-1.09) |  |  |
| VKAs | 7,835 | 117 | 7,323 | 15.98 | 1.00 (reference) | 1.00 (reference) |  |  |
| **Multiple imputation^***^** |  |  |  |  |  |  | NA |  |
| **CPRD** |  |  |  |  |  |  |  |  |
| DOACs | 7,896 | 49 | 6,369 | 7.69 | 0.94 (0.64-1.39) | 0.94 (0.64-1.38) |  |  |
| VKAs | 6,791 | 54 | 7,243 | 7.46 | 1.00 (reference) | 1.00 (reference) |  |  |
| **Intention-to-treat** |  |  |  |  |  |  | 0.81 (0.69-0.96) | 64% |
| **CPRD** |  |  |  |  |  |  |  |  |
| DOACs | 7,896 | 76 | 6,800 | 11.18 | 1.04 (0.75-1.45) | 1.03 (0.75-1.42) |  |  |
| VKAs | 6,791 | 69 | 6,533 | 10.56 | 1.00 (reference) | 1.00 (reference) |  |  |
| **RAMQ** |  |  |  |  |  |  |  |  |
| DOACs | 21,175 | 290 | 18,753 | 15.46 | 0.61 (0.51-0.73) | 0.75 (0.62-0.91) |  |  |
| VKAs | 8,573 | 196 | 7,747 | 25.30 | 1.00 (reference) | 1.00 (reference) |  |  |
| **IPCW**^****^ |  |  |  |  |  |  | 0.92 (0.77-1.10) | 0% |
| **CPRD** |  |  |  |  |  |  |  |  |
| DOACs | 7,896 | 49 | 6,369 | 7.69 | 0.94 (0.64-1.39) | 0.88 (0.60-1.30) |  |  |
| VKAs | 6,791 | 54 | 7,243 | 7.46 | 1.00 (reference) | 1.00 (reference) |  |  |
| **RAMQ** |  |  |  |  |  |  |  |  |
| DOACs | 21,175 | 377 | 32,408 | 11.63 | 0.70 (0.58-0.84) | 0.93 (0.76-1.14) |  |  |
| VKAs | 8,573 | 155 | 8,203 | 18.90 | 1.00 (reference) | 1.00 (reference) |  |  |

^*^ IR per 1,000 PY.

^**^ The study period for this analysis ended on December 31, 2018 due to lack of availability of cause of death in the RAMQ data beyond this date.

^***^ This analysis was conducted only in the CPRD for missing values for body mass index and blood pressure.

^****^ Time-varying covariates included alcohol-related disorders and use of antiplatelet agents.

Abbreviations: DOACs, direct oral anticoagulants; VKAs, vitamin K antagonists; NVAF, non-valvular atrial fibrillation; PY, patient years; IR, incidence rate; HR, hazard ratio; CI, confidence interval; NA, not applicable; IPCW, inverse probability of censoring weighting; CPRD, Clinical Practice Research Datalink; RAMQ, Régie de l'Assurance-Maladie du Québec.

# **eTable 11. Risk of major bleeding associated with use of DOACs compared with use of VKAs among patients with NVAF treated with antiarrhythmics (sensitivity analyses)**

|  | **N**  **Patients** | **N**  **Events** | **N**  **PY** | **IR^*^** | **Crude HR**  **(95% CI)** | **IPTW HR**  **(95% CI)** | **Pooled HR**  **(95% CI)** | **I^2^** |
| --- | --- | --- | --- | --- | --- | --- | --- | --- |
| **15-day grace period** |  |  |  |  |  |  | 0.87 (0.79-0.96) | 61% |
| **CPRD** |  |  |  |  |  |  |  |  |
| DOACs | 7,896 | 179 | 4,164 | 42.99 | 1.09 (0.88-1.35) | 1.01 (0.82-1.25) |  |  |
| VKAs | 6,791 | 164 | 4,324 | 37.93 | 1.00 (reference) | 1.00 (reference) |  |  |
| **RAMQ** |  |  |  |  |  |  |  |  |
| DOACs | 21,175 | 1,305 | 24,042 | 54.28 | 0.68 (0.61-0.75) | 0.84 (0.75-0.93) |  |  |
| VKAs | 8,573 | 511 | 5,434 | 94.04 | 1.00 (reference) | 1.00 (reference) |  |  |
| **Stricter outcome definition** |  |  |  |  |  |  | 0.79 (0.69-0.91) | 0% |
| **CPRD** |  |  |  |  |  |  |  |  |
| DOACs | 7,896 | 128 | 6,291 | 20.35 | 0.84 (0.66-1.06) | 0.79 (0.62-1.00) |  |  |
| VKAs | 6,791 | 164 | 7,108 | 23.07 | 1.00 (reference) | 1.00 (reference) |  |  |
| **RAMQ** |  |  |  |  |  |  |  |  |
| DOACs | 21,175 | 474 | 32,221 | 14.71 | 0.67 (0.56-0.79) | 0.79 (0.67-0.94) |  |  |
| VKAs | 8,573 | 205 | 8,142 | 25.18 | 1.00 (reference) | 1.00 (reference) |  |  |
| **Inclusion of fatal events** |  |  |  |  |  |  | 0.86 (0.79-0.94) | 0% |
| **CPRD** |  |  |  |  |  |  |  |  |
| DOACs | 7,896 | 236 | 6,250 | 37.76 | 0.96 (0.81-1.15) | 0.96 (0.81-1.15) |  |  |
| VKAs | 6,791 | 259 | 7,045 | 36.76 | 1.00 (reference) | 1.00 (reference) |  |  |
| **RAMQ^**^** |  |  |  |  |  |  |  |  |
| DOACs | 16,103 | 1,096 | 20,290 | 54.02 | 0.68 (0.62-0.75) | 0.86 (0.77-0.95) |  |  |
| VKAs | 8,213 | 634 | 7,369 | 86.03 | 1.00 (reference) | 1.00 (reference) |  |  |
| **Exclusion of prior events** |  |  |  |  |  |  | 0.83 (0.76-0.91) | 31% |
| **CPRD** |  |  |  |  |  |  |  |  |
| DOACs | 7,660 | 214 | 6,093 | 35.12 | 0.998 (0.83-1.20) | 0.92 (0.76-1.11) |  |  |
| VKAs | 6,577 | 228 | 6,864 | 33.22 | 1.00 (reference) | 1.00 (reference) |  |  |
| **RAMQ** |  |  |  |  |  |  |  |  |
| DOACs | 18,763 | 1,256 | 28,399 | 44.23 | 0.68 (0.61-0.76) | 0.81 (0.72-0.90) |  |  |
| VKAs | 7,123 | 489 | 6,719 | 72.78 | 1.00 (reference) | 1.00 (reference) |  |  |
| **Multiple imputation^***^** |  |  |  |  |  |  | NA |  |
| **CPRD** |  |  |  |  |  |  |  |  |
| DOACs | 7,896 | 236 | 6,250 | 37.76 | 0.97 (0.81-1.16) | 0.95 (0.79-1.13) |  |  |
| VKAs | 6,791 | 256 | 7,045 | 36.34 | 1.00 (reference) | 1.00 (reference) |  |  |
| **Intention-to-treat** |  |  |  |  |  |  | 0.92 (0.85-1.00) | 0% |
| **CPRD** |  |  |  |  |  |  |  |  |
| DOACs | 7,896 | 276 | 6,708 | 41.14 | 1.00 (0.84-1.18) | 0.96 (0.81-1.14) |  |  |
| VKAs | 6,791 | 262 | 6,436 | 40.71 | 1.00 (reference) | 1.00 (reference) |  |  |
| **RAMQ** |  |  |  |  |  |  |  |  |
| DOACs | 21,175 | 1,221 | 18,272 | 66.82 | 0.72 (0.66-0.79) | 0.91 (0.83-1.01) |  |  |
| VKAs | 8,573 | 691 | 7,483 | 92.34 | 1.00 (reference) | 1.00 (reference) |  |  |
| **IPCW^****^** |  |  |  |  |  |  | 0.91 (0.84-0.99) | 0% |
| **CPRD** |  |  |  |  |  |  |  |  |
| DOACs | 7,896 | 236 | 6,250 | 37.76 | 0.97 (0.81-1.16) | 0.93 (0.77-1.11) |  |  |
| VKAs | 6,791 | 256 | 7,045 | 36.34 | 1.00 (reference) | 1.00 (reference) |  |  |
| **RAMQ** |  |  |  |  |  |  |  |  |
| DOACs | 21,175 | 1,569 | 31,393 | 49.98 | 0.67 (0.61-0.74) | 0.91 (0.82-1.00) |  |  |
| VKAs | 8,573 | 664 | 7,881 | 84.26 | 1.00 (reference) | 1.00 (reference) |  |  |

^*^ IR per 1,000 PY.

^**^ The study period for this analysis ended on December 31, 2018 due to lack of availability of cause of death in the RAMQ data beyond this date.

^***^ This analysis was conducted only in the CPRD for missing values for body mass index and blood pressure.

^****^ Time-varying covariates included alcohol-related disorders, use of antiplatelet agents, and use non-steroidal anti-inflammatory drugs.

Abbreviations: DOACs, direct oral anticoagulants; VKAs, vitamin K antagonists; NVAF, non-valvular atrial fibrillation; PY, patient years; IR, incidence rate; HR, hazard ratio; CI, confidence interval; NA, not applicable; IPCW, inverse probability of censoring weighting; CPRD, Clinical Practice Research Datalink; RAMQ, Régie de l'Assurance-Maladie du Québec.
